# Estimating case fatality risk of severe Yellow Fever cases: systematic literature review and meta-analysis

**DOI:** 10.1186/s12879-021-06535-4

**Published:** 2021-08-16

**Authors:** Joseph L. Servadio, Claudia Muñoz-Zanzi, Matteo Convertino

**Affiliations:** 1 Division of Environmental Health Sciences, University of Minnesota School of Public Health, 420 Delaware St SE, Minneapolis, 55401 MN USA; 2 Nexus Group and Gi-CORE, Graduate School of Information Science and Technology, Hokkaido University, Sapporo, Hokkaido Japan; 3 Institute of Environment and Ecology, Tsinghua Shenzhen International Graduate School, Tsinghua University, Shenzhen, China

**Keywords:** Yellow Fever, case fatality risk, systematic review, meta-analysis

## Abstract

**Background:**

Case fatality risk (CFR), commonly referred to as a case fatality ratio or rate, represents the probability of a disease case being fatal. It is often estimated for various diseases through analysis of surveillance data, case reports, or record examinations. Reported CFR values for Yellow Fever vary, offering wide ranges. Estimates have not been found through systematic literature review, which has been used to estimate CFR of other diseases. This study aims to estimate the case fatality risk of severe Yellow Fever cases through a systematic literature review and meta-analysis.

**Methods:**

A search strategy was implemented in PubMed and Ovid Medline in June 2019 and updated in March 2021, seeking reported severe case counts, defined by fever and either jaundice or hemorrhaging, and the number of those that were fatal. The searches yielded 1,133 studies, and title/abstract review followed by full text review produced 14 articles reporting 32 proportions of fatal cases, 26 of which were suitable for meta-analysis. Four studies with one proportion each were added to include clinical case data from the recent outbreak in Brazil. Data were analyzed through an intercept-only logistic meta-regression with random effects for study. Values of the I^2^ statistic measured heterogeneity across studies.

**Results:**

The estimated CFR was 39 % (95 % CI: 31 %, 47 %). Stratifying by continent showed that South America observed a higher CFR than Africa, though fewer studies reported estimates for South America. No difference was seen between studies reporting surveillance data and studies investigating outbreaks, and no difference was seen among different symptom definitions. High heterogeneity was observed across studies.

**Conclusions:**

Approximately 39 % of severe Yellow Fever cases are estimated to be fatal. This study provides the first systematic literature review to estimate the CFR of Yellow Fever, which can provide insight into outbreak preparedness and estimating underreporting.

**Supplementary Information:**

The online version contains supplementary material available at 10.1186/s12879-021-06535-4.

## Background

Evaluations of infectious disease severity often account for both morbidity and mortality. The latter can be represented by the case fatality risk (CFR), defined as the probability of a case of a disease being fatal [1, 2]. It is, in its simplest form, estimated by a quotient of the number of fatal cases and the total number of cases, which has been reported for disease outbreaks [3–5]. The CFR can be used in these contexts to understand the severity of a disease and implement appropriate policy in the event of an outbreak [6].

Case fatality risks have also been described as case fatality ratios or case fatality rates without difference in definition [7, 8]. Case fatality risk can differ from case fatality rate in that the case fatality risk does not explicitly specify a time period, whereas a case fatality rate implies a period of time [2, 9]. It is common, however, for the three terms to be used interchangeably, regardless of whether time periods are taken into consideration [9].

An estimate for the CFR of a disease based on observed fatal cases is typically included when reporting results from an outbreak investigation [4, 10]. Outbreak investigations for various diseases tend to report the CFR during the time period of observation, though this is not often their primary study aim. Other works aiming to estimate CFR have done so by observing hospital records for the proportion of fatal cases [11] or by tracking outcomes for confirmed cases of diseases [7, 12]. Many of these studies aimed not only to estimate CFR for various diseases, but also to examine risk factors for fatality in order to identify individuals most at risk [8, 11] or examine changes in CFR over time [13]. While valuable for the certainty of information among those recruited, such studies can experience limitations such as ascertaining only the most severe cases or not observing fatal cases who died after data collection [14].

In ascertaining only the most severe cases, the denominator used to calculate the CFR is not representative of all cases. If more severe cases are more likely to be seen in the denominator of the CFR calculation, then the CFR will likely be overestimated due to the denominator representing a subset of cases. Another limitation seen in studies reporting CFRs is the use of unconfirmed cases. Reported CFRs of suspected cases without confirmation may lead to a CFR for a disease that also included non-cases in its calculation. Depending on the proportion of such cases that are fatal, this could lead to an overestimate or an underestimate of the CFR.

Other studies have aimed to estimate CFR by collecting data through literature review [1, 15] and, in many cases, meta-analysis. Some articles have estimated an overall CFR by pooling numerators and denominators across studies [16], pooling study results with a random effects meta analytic method [17–19], or generalized linear models [20], though not all reviews combined results [21].

Yellow Fever (YF), a *Flavivirus* which is spread by multiple genera of mosquito [22], is endemic in sub-Saharan Africa and South America [23], with the ability for even higher burden worldwide due to increased global travel and reemerging outbreaks [24, 25]. An estimated 200,000 global cases are seen per year, with reportedly high case fatality [26]. Future outbreaks of YF have potential to cause major morbidity and mortality; a review evaluating the reproductive number of YF estimated a reproductive number of approximately 4.2, with other estimates ranging between one and 11 [27]. The disease is asymptomatic in a large number of cases, and symptomatic cases present with flu-like symptoms such as fever and body aches [23]. In a smaller number of cases, severe disease develops within a few days, with more severe symptoms including jaundice and hemorrhaging. Estimates of fatality in severe cases commonly used in reports or cited in publications include 50 % [23], 30–60 % [28], and 20–50 % [26]. However, little work exists aiming to evaluate or update these estimates. A 2014 study by Johansson et al. aimed to estimate the proportions of cases that are asymptomatic, mild, and severe, and also estimated a CFR among severe cases which aligned with the WHO estimate of 50 % [15]. This was done through a literature review, where studies were selected through expert knowledge rather than a systematic search strategy [15]. No other works exist that offer an update of this estimate, and none use a systematic method for a literature review.

This study aims to estimate the CFR among severe YF cases through a systematic literature review [29] and meta-analysis. Through literature review, articles were found that contained denominators of severe YF cases and numerators of those cases that were fatal. The CFR was estimated among all studies and then stratified by continent, symptom definitions, and study type, which was based on whether an outbreak investigation was described or cases were reported without known data collection methods. The results of this study offer an estimate of the CFR for severe YF using a comprehensive search of relevant literature.

In conducting the literature review, parts of the search strategy aimed to estimate not only the proportion of fatal cases, but also the proportion of severe and mild cases to estimate burden of disease through proportions of asymptomatic, mild, and severe cases. There were insufficient studies for reliable estimates for disease burden; only methods and results for estimating the CFR among severe cases are presented here.

## Methods

A systematic literature review [29] was conducted to collect relevant data to estimate the CFR of severe YF cases. Severe cases in this study are defined as cases that present with fever along with either jaundice or hemorrhaging, which is consistent with the World Health Organization’s definition [30]. The aim of the literature review was to find articles containing proportions of observed severe YF cases that are fatal, including a numerator and denominator.

### Literature review

This study adhered to the Preferred Reporting Items for Systematic reviews and Meta-Analyses (PRISMA) guidelines [31]. The PRISMA checklist [32] for this review can be found in Additional file 1: Table S1.

The search strategy, as run in PubMed, was as follows: (“Yellow Fever” in the title, abstract, or a medical subject heading) AND (fatal*, severe, severit*, mortality, asymptomatic, symptomatic, diagnosis, misdiagnosis, outbreak, or cases in multiple places) AND NOT (“Vaccine” in title or abstract) with asterisks denoting wildcards. “Vaccine” was excluded because of the high number of studies that aimed to study vaccine efficacy or describe vaccination campaigns, which are outside the aims of this study. A selection of studies excluded due to the “vaccine” criterion were examined and found not to lie within the scope of the analyses for the study aims. The search strategy was run in June 2019, and yielded a total of 485 articles from Ovid Medline and 605 articles from PubMed. After removing duplicates, 842 unique articles underwent title and abstract screening. The search strategy was rerun in March 2021 to add recent studies published between January 2019 and February 2021; 291 articles underwent title and abstract screening from this update.

Articles found via the database search were screened by title and abstract to remove those without relevant information. Articles were excluded if the topic was a disease other than YF or if the title and abstract did not mention investigating or reporting cases of YF. Excluded articles primarily focused on other diseases, laboratory diagnosis methods, or vaccine efficacy research. Following title and abstract screening, 164 articles remained for full text review from the original search, and 47 articles remained for full text review from the updated search.

In full text review, remaining articles were screened for relevant information for the purpose of this study. Articles were included if full text was available in English and contained both a denominator of total severe YF cases and a numerator of deaths among the severe cases. Articles that did not report a numerator and denominator, but did report a denominator and fatality proportion for YF cases, were included. While some studies reported that YF cases were laboratory confirmed, others did not report laboratory confirmation and classified identified YF cases by symptoms. Articles that were not included most commonly included editorials, single case reports, reporting of cases in nonhuman primates, reports of capacity building, or laboratory detection methods. Of the articles undergoing full text review from the original search, 14 were not available in full text, seven were not available in English, and 117 focused on YF topics, but did not include a specific denominator of cases or numerator of fatal cases. During full text review from the original search, four articles were added from the references of the articles read, yielding 30 articles in total. From the updated search, one article was retained from full text review, and another was added from references of the 47 articles. Following these steps, the 32 total articles were reread to validate the data extracted.

**Fig. 1:**
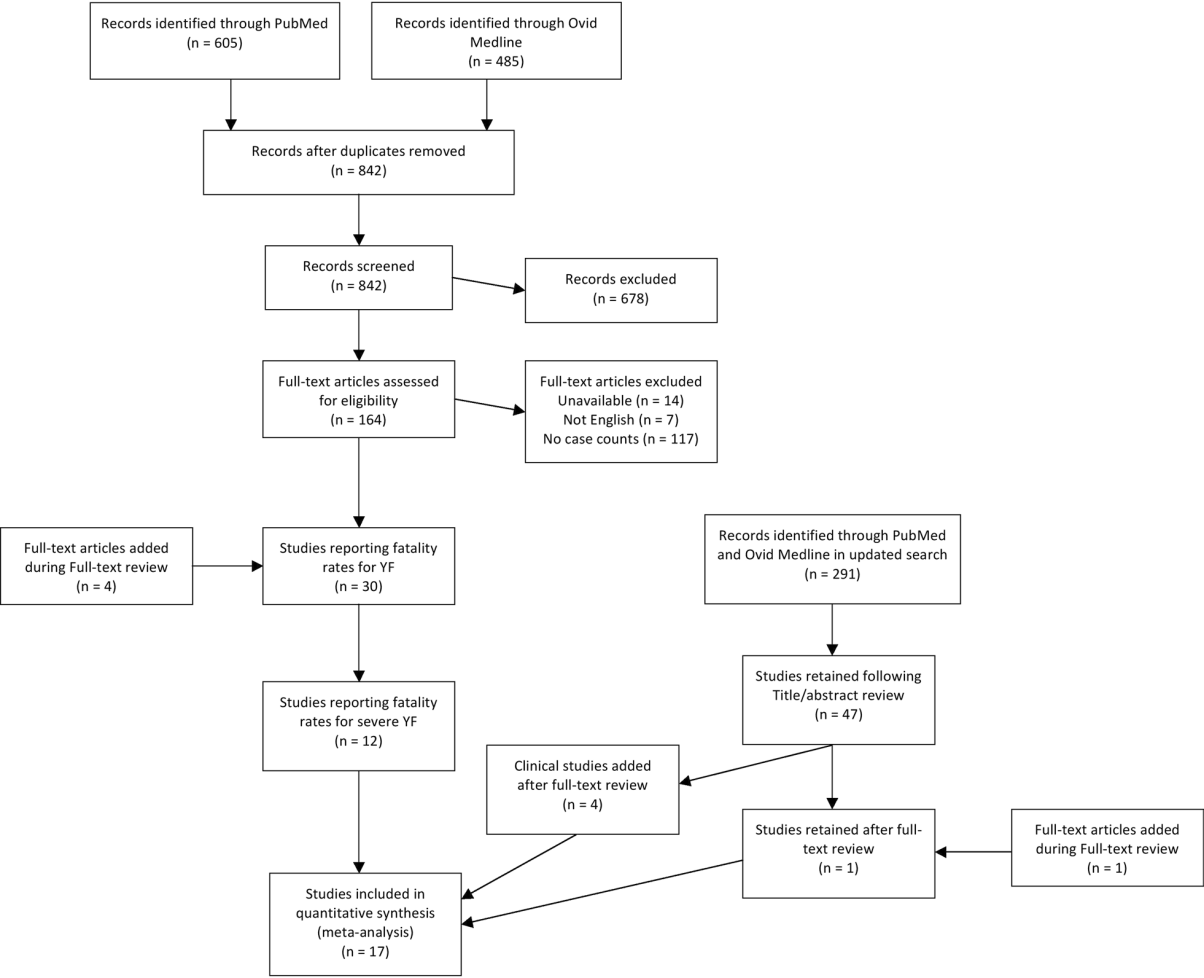
Flow diagram for screening and including articles in systematic literature review and meta-analysis for case fatality risk of severe Yellow Fever cases. Publication years of articles ranged between 1972 and 2020

Following data extraction from the 32 articles, those that did not specifically report severe cases were removed. In this study, severe YF cases were defined by having a fever and at least one of jaundice or hemorrhaging. Studies that did not state that YF cases were defined by having fever as well as at least one of jaundice of hemorrhaging were removed. During the updated search, this criterion was applied during full text review. These symptoms were typically reported at the study level, where the authors of the studies stated broadly the symptoms used in identifying YF cases in the main text. Many studies only included fever and jaundice in the definition of severe YF, while others included hemorrhaging and other symptoms including organ failure (Additional file 2: Table S2). A total of 14 studies contained explicit numbers for fatal and nonfatal severe cases through these definitions (Fig. 1; Table 1). In order to incorporate data from the recent YF outbreak in Brazil, four studies from the updated search that specified severe YF cases through healthcare use, but did not explicitly state the symptom definition outlined previously as inclusion criteria, were added. These studies stated that some patients showed symptoms such as fever, jaundice, and hemorrhaging, but the numbers of fatal and total cases did not exclusively consist of cases meeting the symptom definition.

**Table. 1:** Studies found through systematic literature review for case fatality risk among severe Yellow Fever cases. Paper numbers above 1000 were added when updating the literature search. Paper numbers above 2000 were added outside the literature search. Studies with a numerator equal to zero were excluded from meta-analysis

Paper Number	Authors	Country	Year	Lab confirmed diagnosis	Study Type	Numerator	Denominator
1	WHO 2008 [36]	Cote d’Ivoire	2006	Yes	Report	3	16
1	WHO 2008 [36]	Mali	2006	Yes	Report	4	5
1	WHO 2008 [36]	Cameroon	2006	Yes	Report	0	1
1	WHO 2008 [36]	Central African Republic	2006	Yes	Report	0	1
1	WHO 2008 [36]	Guinea	2006	Yes	Report	0	1
1	WHO 2008 [36]	Ghana	2006	Yes	Report	0	1
21	Wamala 2012 [37]	Uganda	2010–2011	Yes	Investigation	45	181
22	de Filippis 2002 [38]	Brazil	2002	Yes	Investigation	17	32
48	Tuboi 2007 [39]	Brazil	1998–2003	Yes	Report	111	251
48	Tuboi 2007 [39]	Brazil	1998–2007	Yes	Report	67	97
81	Jones 1972 [40]	Nigeria	1969	Yes	Investigation	47	103
122	WHO 1993 [41]	Kenya	1993	Yes	Report	18	41
167	Ingelbeen 2018 [42]	Democratic Republic of Congo	2017	Yes	Report	18	78
170	Monath 1980 [43]	Gambia	1978–1979	Yes	Investigation	13	67
154	WHO 2011 [44]	Cameroon	2009	Yes	Report	0	1
73	Agadzi 1984 [45]	Ghana	1977–1978	No	Investigation	34	136
73	Agadzi 1984 [45]	Ghana	1978	No	Investigation	12	32
73	Agadzi 1984 [45]	Ghana	1978–1979	No	Investigation	44	207
73	Agadzi 1984 [45]	Ghana	1978	No	Investigation	53	340
73	Agadzi 1984 [45]	Ghana	1979	No	Investigation	41	104
73	Agadzi 1984 [45]	Ghana	1980	No	Investigation	6	8
168	deCock 1988 [46]	Nigeria	1986	No	Investigation	59	126
154	WHO 2011 [44]	Congo	2009	No	Report	0	1
171	Nasidi 1989 [47]	Nigeria	1987	No	Investigation	202	325
171	Nasidi 1989 [47]	Nigeria	1987	No	Investigation	192	255
171	Nasidi 1989 [47]	Nigeria	1987	No	Investigation	416	805
171	Nasidi 1989 [47]	Nigeria	1987	No	Investigation	8	72
171	Nasidi 1989 [47]	Nigeria	1987	No	Investigation	3	9
171	Nasidi 1989 [47]	Nigeria	1987	No	Investigation	6	7
171	Nasidi 1989 [47]	Nigeria	1987	No	Investigation	9	69
1001	Cunha 2019 [48]	Brazil	2018	Yes	Reporting	176	498
1002	Otshuniema 2017 [49]	Democratic Republic of Congo	2016	Yes	Reporting	8	37
2001	de Avila 2020 [50]	Brazil	2017–2018	Yes	Investigation	54	114
2002	Ribeiro 2019 [51]	Brazil	2018	Yes	Investigation	21	72
2003	Ho 2019 [52]	Brazil	2018	Yes	Investigation	53	79
2004	Kallas 2019 [53]	Brazil	2018	Yes	Investigation	27	76

In addition to the numbers of total and fatal severe cases, descriptive information was collected for each study. Data collected included country, continent, year, symptoms, applied case definitions, and research methods for each study. Case definition and symptoms in the main text were used to confirm that cases listed were severe cases. While, for the purposes of this study, fever as well as either jaundice or hemorrhaging were required for inclusion as severe YF cases, some included studies considered other symptoms as well in their case definitions, including abdominal pain and organ failure (Additional file 2: Table S2). It also was noted whether the authors were active in recruiting YF patients or stating numbers from reported case data. If the authors reported specifying a case definition, being present in data collection, or stating details of an outbreak investigation, then the study was classified as an “investigative” study. Reports in which case counts were reported without specifically describing active measures to identify cases were classified as “reporting” studies. All studies were classified as either an investigative or reporting study (Table 1).

Also collected was whether the cases in each study were confirmed or suspected for YF. Cases were considered confirmed if the articles stated laboratory diagnostic confirmation for YF. Studies that specified a specific laboratory diagnosis, such as through polymerase chain reaction (PCR), or stated lab confirmed cases without specifying a specific type of test, were included as laboratory confirmed cases. Cases were considered suspect if the article explicitly stated cases were suspect. Probable cases, where symptoms are observed without a guaranteed laboratory result, were considered suspect cases in this study. If the article did not state whether cases were confirmed or suspect, then cases were assumed to be suspect (Table 1).

All included studies list total and fatal counts of severe YF cases without the CFR being the primary focus of the study. Therefore, the sources of bias in the individual studies remained consistent. Two possible major sources of bias are underreporting of cases within studies and deaths occurring after followup [14]. Underreported cases, if nonfatal, would lead to a smaller denominator and an overestimated CFR. If the underreported cases were as fatal as reported cases, no bias would be observed. Deaths occurring after followup would lead to fatal cases being classified as nonfatal, and lead to an underestimate of CFR.

Despite these two potential sources of bias, publication bias, where tests of statistical significance affect reporting of results [33], is not likely to be present in this study. Because the primary focus of the studies was not to estimate the CFR of YF, issues of publication bias are less likely since no included articles used statistical significance tests for CFR estimates. Therefore, the proportions of fatal cases within studies are unlikely to affect whether the studies were published.

Collected data were inputted into a Microsoft Excel (2013) spreadsheet during full text review, with information to be collected determined prior to data collection.

#### Data analysis

Case fatality risk was estimated using a meta-analysis for proportions. The metaprop() function from the ‘meta’ R package [34] was used to run an intercept-only logistic meta-regression with random effects for study. Model inputs included the observed proportion of fatal cases for each study, $\widehat{p}$, as well as its standard error, estimated by $\sqrt{\frac{\widehat{p}(1- \widehat{p})}{n}}$, where $n$ is the denominator of the study. Only proportions not equal to zero or one were included in the meta-analysis, as these would produce standard errors of zero. They are, however, included in Table 1. Estimates for CFR were found for laboratory confirmed, suspected, and all severe YF cases. Stratified CFRs were estimated for differences by continent (South America or Africa), by study type (investigative or reporting, as defined previously) and by symptoms reported (fever and jaundice or fever, jaundice, and other severe symptoms). Values of the I^2^ statistic were calculated to describe heterogeneity [35]. Analyses were run separately to include and exclude the four recent studies added to show whether results are sensitive to the inclusion of studies containing data likely to be useful, but not meeting the strict inclusion criteria.

## Results

### Article inclusion

A total of 18 studies were found through the literature review reporting a CFR for severe YF, three of which were added from the references of the 211 articles that underwent full-text screening and four of which underwent full text screening and were included due to their relevance to the recent Brazilian outbreak. The 18 papers contained a total of 36 proportions of fatal severe cases; 30 of these, present in 17 studies, were not equal to zero or one (Table 1) and therefore included in the meta-analysis. The six proportions that equaled zero or one were instances where only one severe YF case was reported in the denominator. Of the 30 proportions included in the meta-analysis, 14 articles provided 16 proportions of CFR among confirmed severe cases of YF, and another three articles provided 14 proportions among severe suspected cases.

**Fig. 2:**
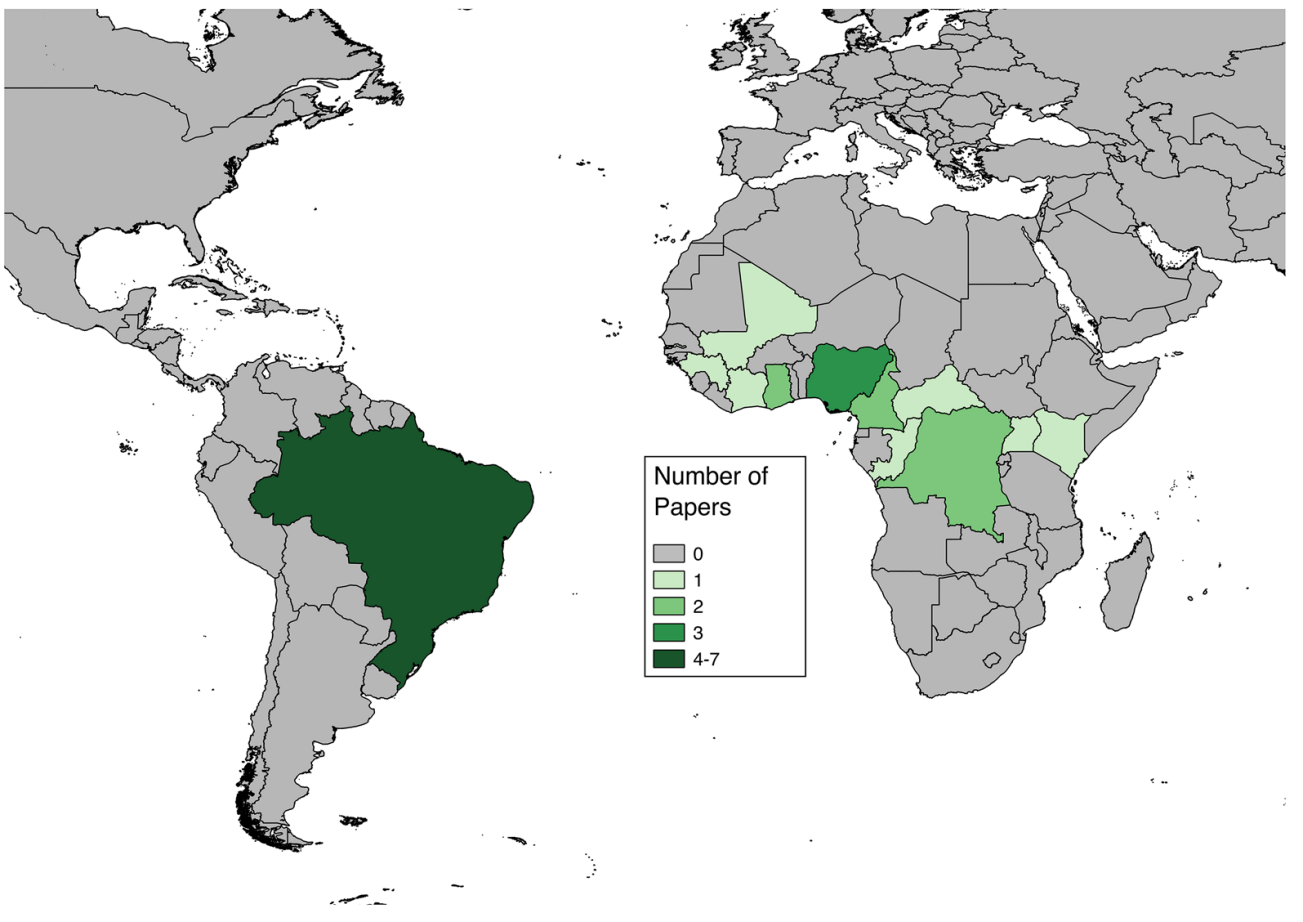
Numbers of articles found through systematic literature review reporting case fatality risk data for severe Yellow Fever cases for each nation. Some articles contained data for multiple nations

**Fig. 3:**
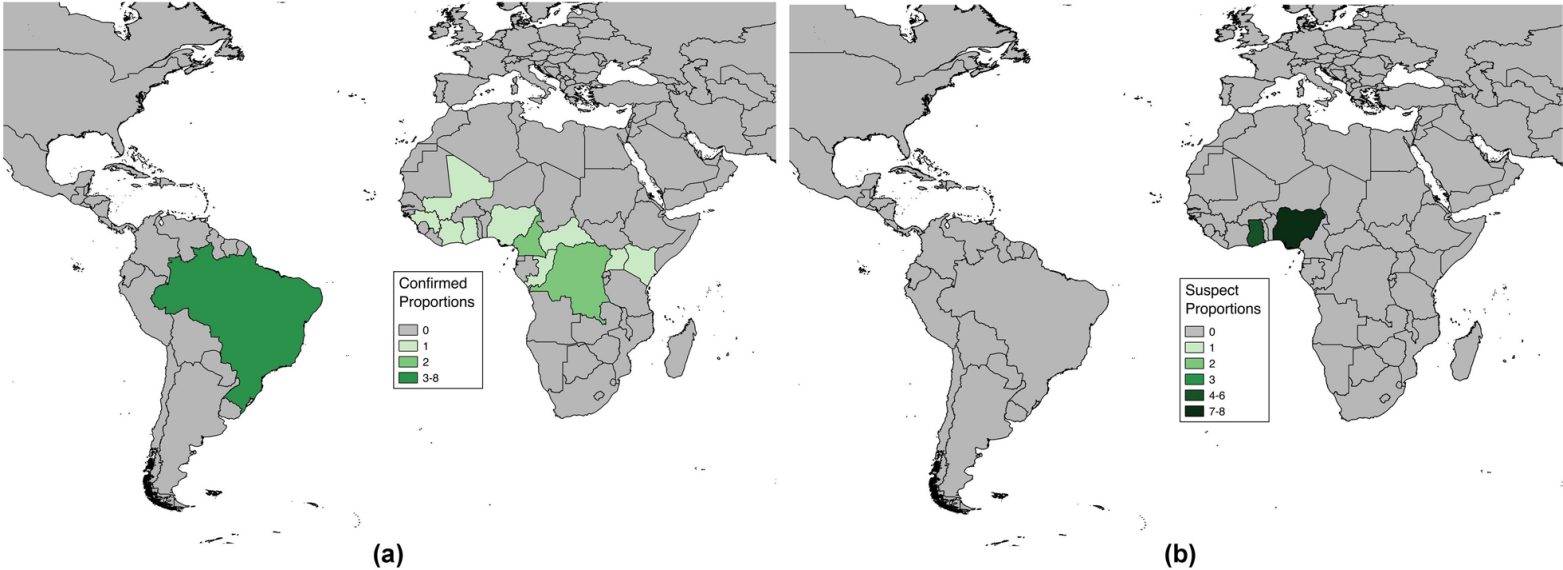
Numbers of proportions for case fatality risk among severe Yellow Fever cases found through systematic literature review by nation. Numbers of proportions are separated by (**a**) confirmed and (**b**) suspect severe Yellow Fever cases

Articles included in analyses reported cases in both Africa and South America (Fig. 2). The countries with more than one study or more than one fatality proportion found through the literature review were Brazil (7 papers, 8 proportions), Nigeria (3 papers, 9 proportions), Ghana (1 paper, 6 proportions), Cameroon (2 papers, 2 proportions), and Democratic Republic of Congo (2 papers, 2 proportions) (Fig. 3). Articles reporting multiple proportions in the same country during the same year(s) reported different proportions for different locations within countries.

Articles from the initial search ranged in year between 1942 and 2019, with 544 (64 %) published in 2000 or after. Articles from the updated search ranged between 2019 and 2021, with the article added from references in full text review, which did not appear in the initial search, published in 2017. Among the 18 final articles with severe YF fatality proportions, publication years ranged between 1972 and 2020, with 12 (66 %) published in 2000 or after (Table 1). Of these, four papers are clustered between 1984 and 1993, another four are clustered between 2007 and 2012, seven are clustered between 2017 and 2020, and the remaining three are interspersed outside these time clusters.

Among the 36 fatality proportions for severe YF found in literature, 21 represented fatality proportions among laboratory confirmed severe cases (Table 1), though the laboratory test used was not always specified in the text. The remaining 15 represented proportions from suspect severe cases, where laboratory confirmation was not stated. From assessing the study methods, 22 proportions were found from investigative studies as described previously, with the remaining 14 presented in reporting studies. All proportions, with the exception of those from recent clinical investigations in Brazil, explicitly stated use of both fever and jaundice in their case definitions, and some studies also included other symptoms as well in YF case diagnosis. These included hemorrhaging (15 proportions), abdominal pain (13 proportions), or organ failure (2 proportions) (Additional file 2: Table S2). Travel to a YF endemic region was a criterion included in eight proportions.

### Estimates of CFR

**Fig. 4:**
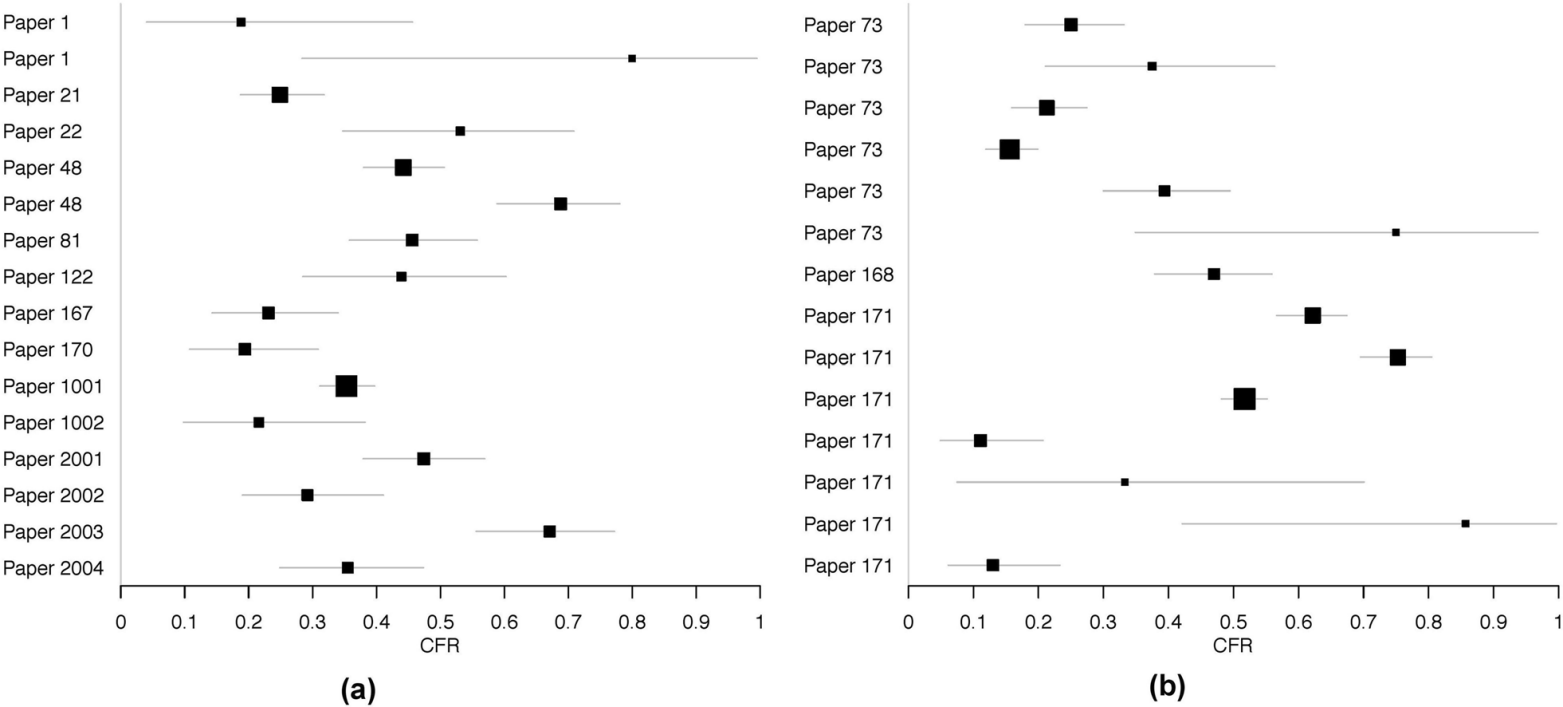
Forest plots of case fatality risk estimates among severe (**a**) laboratory confirmed and (**b**) suspect Yellow Fever cases found through literature review. Only risk estimates not equal to zero are included

Forest plots are shown in Fig. 4, separated by confirmed and suspect YF cases. The individual numerators and denominators in each study are shown in Table 1.

The estimated CFR among all severe cases was 39 %, with a 95 % confidence interval of [31 %, 47 %]. Separating CFR by case confirmation yielded no substantial differences in CFR. Including or excluding the four recent clinical studies from Brazil also did not lead to substantial differences in CFR estimates (Table 2). Much heterogeneity was seen among studies, as indicated by I^2^ values.

**Table. 2:** Estimated case fatality risk among severe Yellow Fever cases with 95% confidence intervals, I^2^ statistic values, and numbers of proportions analyzed

	Confirmed	Suspected	Combined
Without added studies	37 % [28 %, 47 %] I^2^ = 0.91 n = 12	39 % [26 %, 53 %] I^2^ = 0.97 n = 14	38 % [30 %, 47 %] I^2^ = 0.96 n = 26
With clinical studies	39 % [31 %, 48 %] I^2^ = 0.90 n = 16	39 % [26 %, 53 %] I^2^ = 0.97 n = 14	39 % [31 %, 47 %] I^2^ = 0.95 n = 30

### Stratified CFR

The CFRs for severe YF cases were stratified by characteristics of the articles to account for potential heterogeneities in either YF dynamics or data collection and reporting. First, CFRs were stratified by continent. Combining proportions of both confirmed and suspected severe cases, the estimated CFR among severe cases in African countries is 36 % (95 % CI: [27 %, 45 %], n = 22), and the estimated CFR among South American countries is 47 % (95 % CI: [38 %, 57 %], n = 8). Among investigative studies, the estimated CFR was 39 % (95 % CI: [30 %, 48 %], n = 22), and among reporting articles, the estimated CFR was 39 % (95 % CI: [27 %, 53 %], n = 8). Among studies that reported severe cases with only fever and jaundice, the estimated CFR was 38 % (95 % CI: [24 %, 53 %], n = 13), and among studies with cases showing other symptoms beyond fever and jaundice, the estimated CFR was 39 % (95 % CI: [32 %, 48 %], n = 17).

## Discussion

This study aimed to systematically evaluate the CFR for severe YF cases, defined as cases showing fever with either jaundice or hemorrhaging. A systematic literature review was conducted in order to find reported proportions of fatal cases of severe YF. Using 30 proportions recorded from 17 articles in a meta-analysis, the estimated CFR was 39 %, which was consistent among both confirmed and suspected severe cases. Separating the CFR by continent showed a notably higher CFR in South America compared to Africa, separating the CFR among severe cases by article type showed no difference in CFR between investigative studies and passively reported cases, and separating by inclusion of symptoms beyond fever and jaundice showed no difference in CFR estimates. The estimated CFR of 39 % is lower than the common estimate of half of severe cases being fatal [23] but consistent with other fatality ranges reported [26, 28]. While there is uncertainty around all estimates, a difference of approximately 10 percentage points between this study’s estimate and the statement of half of cases being fatal may be notable for clinical decision-making and perceived mortality of YF.

The drastic difference in estimated CFR between South America and Africa may potentially result from differences in data collection as well as differences in YF dynamics. Other systematic reviews estimating CFR have seen geographic differences, including separating Hong Kong and other regions from mainland China in estimating CFR for hand foot and mouth disease [54] and separating WHO world regions to stratify CFR for *Salmonella* infections [55]. The differences seen across geographic locations in these studies were less pronounced than the difference in CFR between continents found in this study.

Different strains of YF are found between South America and Africa [56, 57] as well as different primary mosquito vectors and nonhuman primate reservoirs [58]. Clinical care also differs across countries. Though International Health Regulations require reporting of YF cases [59], implementation and surveillance quality may differ between the two continents, as well as differences in healthcare seeking behaviors, which can lead to differences in severe cases represented across each continent. The small number of proportions representing CFR in South America may also account for the difference in estimated CFR across continents, as having eight South American proportions makes the CFR estimate more sensitive to any single study providing a non-representative sample of severe YF cases.

Across studies, there was significant heterogeneity among the CFRs reported (Fig. 4; Table 2). Among other reviews estimating CFR for other infectious diseases, high heterogeneities have also been seen [55, 60, 61]. Potential sources of heterogeneity across the different studies include differences in surveillance resources as well as differences in healthcare infrastructure across the various settings of the studies.

The results of this study show a reported CFR that is notably lower than the estimate of 50 % reported by the WHO [23] and by Johansson et al. [15]. This does not, however, suggest that the estimated CFR from this study will apply to every outbreak situation. The CFRs of other diseases have been seen to change over time [13] and may differ in relation to industrialization [62]. The estimate yielded in this study should be used as an average CFR and broad recommendation.

It is important to note that the interpretation of the estimated CFR in this study is based on the case definition used. This study produced estimates for the CFR among severe YF cases rather than among all YF cases, with severe YF cases defined by symptoms as described previously. A CFR among all YF cases, which would commonly include all symptomatic cases beyond the definition imposed in this study, would be lower. Further, an estimate of the infection fatality ratio (IFR) would represent risk of fatality among all infections, which includes asymptomatic infections, and be even lower than the CFR for all YF cases. The CFR of 39 % should only be applied to YF cases with fever and jaundice or hemorrhaging rather than to all infections or cases outside this definition; severe YF cases comprise approximately 15 % of all YF cases [63], and an even lower percentage of infections, so the IFR would be expected to be notably lower than 39 %. Relaxing the symptomatic definition to include cases of confirmed YF presenting in healthcare settings did not lead to notable differences in CFR estimates (Table 2).

This study’s literature review yielded a total of 18 relevant studies, which provided 36 proportions of fatal severe YF cases, 30 of which were included in meta-analysis. Other studies using systematic review methods to estimate the CFR of other diseases commonly have more available papers relevant to the study aims [16, 55, 60], though others have had similarly lower article counts [54, 61, 64]. Many of these studies also used the I^2^ statistic to consider heterogeneity, with many of them similarly showing high heterogeneity [20–22]. Stratification by geography was also seen in other studies [54, 55].

This study benefits from the use of a comprehensive strategy for literature review, which maximizes the completeness of data available for analysis. Conducting a literature review rather than estimating CFR solely from surveillance data allowed multiple outbreak investigations to contribute to the data analysis. As a result, studies with researchers playing a more active role in surveillance of YF cases, which may have greater accuracy, were included.

This study also stratified CFR by the methods of the individual studies into investigative and reporting studies. Separating the studies by the researchers’ involvement in patient recruitment and assessing symptoms of cases, however, showed no difference in CFR between studies with researchers involved in the investigation and studies reporting surveillance statistics. Prior to updating the search strategy, however, studies reporting surveillance statistics had a higher CFR (44 %, 95 % CI: [28 %, 61 %]). Both types of studies could experience different limitations to accuracy. Following the updated search strategy results, the similarity between the two estimates can demonstrate consistency, and potentially validity, in these two types of surveillance.

The analyses in this study included CFR estimates for confirmed and suspected severe YF cases separately as well as combined. Because YF can present similar symptoms and be misdiagnosed for other diseases such as dengue [65], there is less certainty of whether suspected YF cases in this study are true YF cases. However, the similarity in stratified results comparing laboratory confirmed and suspected YF cases shows that excluding the suspected cases from analyses would not lead to a substantial change in conclusions.

### Use for estimating burden

An initial aim of this study was to use the systematic review to also collect data to estimate total cases through estimating proportions of cases that are asymptomatic and mild, similarly to the 2014 study by Johansson et al. [15]. There were insufficient studies from the literature review to reliably generate these estimates due to inconsistency of study results and few studies reporting such information. This is evidence of the challenges inherent to collecting highly detailed data, particularly in less affluent nations, which typically experience higher burdens of YF and other vector-borne diseases. However, having a reliable estimate for CFR, as generated in this study, can prove useful for attempting to quantify underreporting of YF cases. Case reports with higher proportions of fatal cases may suffer from underreporting under the assumption that the CFR found in this study is broadly applicable to other incidence of YF. For example, using data provided from the Pan American Health Organization and the Brazilian Ministry of Health, 157 fatal among 327 confirmed cases of YF were reported via surveillance in Brazil between 2000 and 2014, which may include non-severe cases. Under the assumptions that the 39 % CFR found in this study is applicable to these data, no fatal cases were unreported, and only severe cases become fatal, estimates of actual case counts can be produced. By multiplying the 157 fatal cases by the inverse of the estimated CFR, an estimate of 403 severe YF cases is obtained. If all 327 reported cases were severe cases, then approximately 19 % of severe cases were undetected. This serves as a minimum proportion of underreported cases rather than an estimate [15] since this assumes all fatal cases were observed and all reported cases were severe.

### Limitations and future directions

Through the literature search, 14 studies were identified to fit the defined criteria for severe YF, 13 of which were suitable to be used in meta-analysis. The requirement that studies must indicate that cases present fever as well as either jaundice or hemorrhaging for inclusion in this study led to several studies to be excluded. Many studies stated numbers of cases and fatalities without specifying symptoms [44, 66–71], which included studies from the recent outbreak in Angola [66, 67]. Studies representing cases from the recent YF outbreak in Brazil, though not stating that all cases reported showed the symptoms, were added to the analyses to show whether inclusion of confirmed cases from a clinical setting might impact substantive results [50–53]. While the symptom requirements in this study led to potentially useful sources of information to be excluded from analysis, they do increase confidence that the CFR estimated applies directly to severe YF cases by not including potentially mild cases. Similarity in results when including four studies of YF cases seeking healthcare increase confidence that the results may be more broadly applicable.

The results of this study rely on the reported data from the 17 studies used in the meta-analysis. Because the purpose of these articles was not necessarily to offer estimates of the CFR for YF, it is possible that maximizing the accuracy of fatal and nonfatal case counts was not the highest priority. The studies detailing outbreak investigations do report numbers of severe and fatal cases, but the purpose was not to assure generalizable accuracy of the CFR. This possibility is even stronger among reporting studies. Since underreporting of infectious disease cases is a well established issue [72], it is likely that the proportions used in this analysis may also be subject to issues of data quality.

Within this review, the results are limited by the heterogeneity in studies and the assumption that different world regions are expected to have similar CFRs. In combining the studies across nations in South America and Africa, where stratified CFRs differed notably, it is assumed that the differences observed are artifacts of the individual studies rather than indicative of actual differences in CFR across the two continents. Heterogeneity likely exists within continents as well, as the nations represented in this study include both East and West Africa (Fig. 2). This heterogeneity may result from actual differences in probability of fatality; risk of fatality may differ by population demographics [1, 73] or national industrialization [62], as seen in other diseases.

## Conclusions

Among severe cases of YF, the CFR is estimated to be approximately 39 % based on the results of a systematic literature review. This is lower than the frequently cited CFR for severe cases, indicating that the previous estimate is either a cautious estimate or based on underreported data. However, these results indicate high fatality among severe YF cases, demonstrating the public health importance of this disease. Preventative measures such as vaccination and diagnosis methods are of importance for reducing deaths from YF.

Use of systematic reviews for estimating CFR has been seen for other diseases, and this method can be extended to further characteristics of various diseases beyond CFR. Further research is needed to distinguish among asymptomatic, mild, and severe YF infections in order to most accurately estimate the total burden of disease. The estimate of CFR found in this study can be used to estimate potential mortality in future YF outbreaks.

## Supplementary Information

**Additional file 1: Table S1.** Preferred Reporting Items for Systematic reviews and Meta-Analyses (PRISMA) checklist for systematic review and meta-analysis for case fatality risk of severe Yellow Fever cases.

**Additional file 2: Table S2.** Laboratory confirmation and symptom definitions used by included articles. Marked symptoms were required for case inclusion, “or__” indicates a set of symptoms where at least one from the set was required, and “some” indicates that some, but not all, cases showed the symptom.

## Data Availability

The data analyzed within this study are shown in Table 1.

## Abbreviations

YF: Yellow Fever
CFR: Case fatality risk
PRISMA: Preferred reporting items for systematic reviews and meta-analyses
PCR: Polymerase chain reaction
