# Supplementary material for: Estimating case fatality risk of severe Yellow Fever cases: systematic literature review and meta-analysis
Source: BMC Infect Dis. 2021 Aug 16;21:819. doi: 10.1186/s12879-021-06535-4 (PMC8365934; doi:10.1186/s12879-021-06535-4)
Supplement: Supplementary file 2 — Additional file 2: Table S2. Laboratory confirmation and symptom definitions used by included articles. Marked symptoms were required for case inclusion, “or__” indicates a set of symptoms where at least one from the set was required, and “some” indicates that some, but not all, cases showed the symptom. [file 12879_2021_6535_MOESM2_ESM.docx]

Estimating case fatality risk of severe Yellow Fever cases: Systematic literature review and meta-analysis

Joseph L Servadio^1^*, Claudia Muñoz-Zanzi^1^, Matteo Convertino^2,3^

^1^Division of Environmental Health Sciences, University of Minnesota School of Public Health, Minneapolis, MN, USA

^2^ Nexus Group and Gi-CORE, Graduate School of Information Science and Technology, Hokkaido University, Hokkaido, Sapporo, Japan

^3^ Institute of Environment and Ecology, Tsinghua Shenzhen International Graduate School, Tsinghua University, Shenzhen, China

*Corresponding author: Joseph L Servadio, Division of Environmental Health Sciences, University of Minnesota School of Public Health, 420 Delaware St SE, Minneapolis, MN 55401 USA. serva024@umn.edu.

Table S2. Laboratory confirmation and symptom definitions used by included articles. Marked symptoms were required for case inclusion, “or__” indicates a set of symptoms where at least one from the set was required, and “some” indicates that some, but not all, cases showed the symptom.

| Paper Num | Country | Lab confirmed | Fever | Jaundice | Abdominal pain | Hemorrhaging | Organ Failure | Travel |
| --- | --- | --- | --- | --- | --- | --- | --- | --- |
| 1 | Cameroon | X | X | X |  |  | X |  |
| 1 | Central African Republic | X | X | X |  |  |  |  |
| 1 | Cote d'Ivoire | X | X | X |  |  |  |  |
| 1 | Ghana | X | X | X |  |  |  |  |
| 1 | Guinea | X | X | X |  |  |  |  |
| 1 | Mali | X | X | X |  |  |  |  |
| 21 | Uganda | X | X | or |  | or |  |  |
| 22 | Brazil | X | or | or | or |  |  |  |
| 48 | Brazil | X | X | X | or | or |  | or |
| 48 | Brazil | X | X |  |  | X |  |  |
| 73 | Ghana |  | or1 | or1 | or1 | or1 |  | or2 |
| 73 | Ghana |  | or1 | or1 | or1 | or1 |  | or2 |
| 73 | Ghana |  | or1 | or1 | or1 | or1 |  | or2 |
| 73 | Ghana |  | or1 | or1 | or1 | or1 |  | or2 |
| 73 | Ghana |  | or1 | or1 | or1 | or1 |  | or2 |
| 73 | Ghana |  | or1 | or1 | or1 | or1 |  | or2 |
| 81 | Nigeria | X |  | or | or | or |  |  |
| 122 | Kenya | X | or | or |  | or | or |  |
| 154 | Cameroon | X | X | X |  |  |  |  |
| 154 | Congo |  | X | X | X |  |  |  |
| 167 | Democratic Republic of Congo | X | X | X |  |  |  |  |
| 168 | Nigeria |  | X | X |  |  |  |  |
| 170 | Gambia | X | X | X |  |  |  |  |
| 171 | Nigeria |  | X | X |  |  |  |  |
| 171 | Nigeria |  | X | X |  |  |  |  |
| 171 | Nigeria |  | X | X |  |  |  |  |
| 171 | Nigeria |  | X | X |  |  |  |  |
| 171 | Nigeria |  | X | X |  |  |  |  |
| 171 | Nigeria |  | X | X |  |  |  |  |
| 171 | Nigeria |  | X | X |  |  |  |  |
| 1001 | Brazil | X | X | or |  | or |  |  |
| 1002 | Democratic Republic of Congo | X | X | X |  |  |  |  |
| 2001 | Brazil | X | some | some | some |  |  |  |
| 2002 | Brazil | X | some/X | some/X |  | some |  |  |
| 2003 | Brazil | X | some | some | some | some |  |  |
| 2004 | Brazil | X | some |  | some | some |  | some |
